# A New Emulsion Liquid Membrane Based on a Palm Oil for the Extraction of Heavy Metals

**DOI:** 10.3390/membranes5020168

**Published:** 2015-04-23

**Authors:** Sanna Björkegren, Rose Fassihi Karimi, Anna Martinelli, Natesan Subramanian Jayakumar, Mohd Ali Hashim

**Affiliations:** 1 Applied Surface Chemistry, Chemical and Biological Engineering, Chalmers University of Technology, SE-412 96 Gothenburg, Sweden; E-Mails: fassihi.rose@gmail.com (R.F.K.); anna.martinelli@chalmers.se (A.M.); 2 Department of Chemical Engineering, University of Malaya, Kuala Lumpur 50603, Malaysia; E-Mails: jaya_kumar@um.edu.my (N.S.J.); alihashim@um.edu.my (M.A.H.)

**Keywords:** emulsion liquid membrane (ELM), palm oil, hexavalent chromium, ionic liquid, green chemistry

## Abstract

The extraction efficiency of hexavalent chromium, Cr(VI), from water has been investigated using a vegetable oil based emulsion liquid membrane (ELM) technique. The main purpose of this study was to create a novel ELM formulation by choosing a more environmentally friendly and non-toxic diluent such as palm oil. The membrane phase so formulated includes the mobile carrier tri-*n*-octylmethylammonium chloride (TOMAC), to facilitate the metal transport, and the hydrophilic surfactant Tween 80 to facilitate the dispersion of the ELM phase in the aqueous solution. Span 80 is used as surfactant and butanol as co-surfactant. Our results demonstrate that this novel ELM formulation, using the vegetable palm oil as diluent, is useful for the removal of hexavalent chromium with an efficiency of over 99% and is thus competitive with the already existing, yet less environmentally friendly, ELM formulations. This result was achieved with an optimal concentration of 0.1 M NaOH as stripping agent and an external phase pH of 0.5. Different water qualities have also been investigated showing that the type of water (deionized, distilled, or tap water) does not significantly influence the extraction rate.

## 1. Introduction

**Figure 1:**
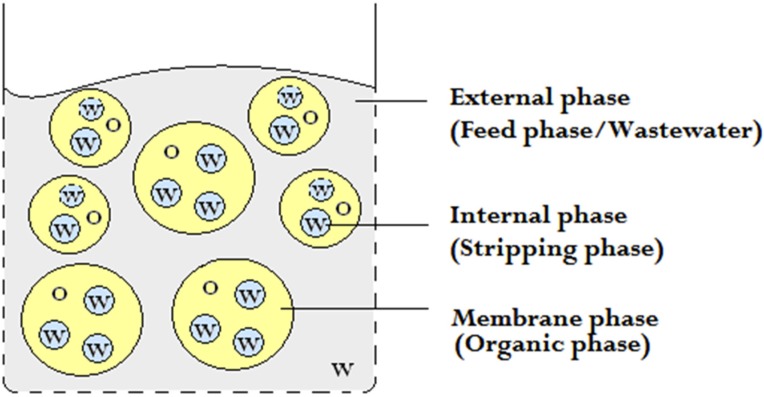
The phases in a water-in-oil-in-water emulsion (w/o/w). O = Oil (Yellow) and W = Water (Gray for external phase and blue for interal phase).

The removal and recovery of heavy metals from wastewater and industrial effluents is environmentally and economically driven as much as it is a health issue. In large parts of the world, such as Southeast Asia, the contamination of groundwater and water resources is a major concern wherefore efficient, economic, and sustainable methods for purification of water are required. An example of a metal that can be found is chromium, which exists in both its trivalent, Cr(III), and hexavalent, Cr(VI), form. Hexavalent chromium is highly poisonous, an oral dose of 2–5 g Cr(VI) can be fatal to an adult human [1]. The World Health Organization (WHO) has a provisional guideline value of 0.05 ppm for the total chromium concentration in drinking water [2]. It is therefore important to purify wastewater before it reaches the environment. The extraction capability of liquid membranes has been used successfully in many areas, such as metal ion extraction, separation of inorganic species, and biochemical and biomedical applications [3]. Although the first patent on liquid membranes was published in 1968 [4], the field of liquid membranes as a separation technique is still expanding in research and in its application as an industrial separation process. Liquid membranes consist of three distinct phases: the feed phase, the membrane phase, and the stripping phase, as schematically shown in Figure 1. Among the different kinds of liquid membranes, e.g., bulk liquid membrane (BLM), supported liquid membrane (SLM), and emulsion liquid membrane (ELM), the double emulsion in ELM results in the highest mass transfer area in addition to a high selectivity and a high metal transfer flux, due to the possibilities of incorporating chemical components that enhance the metal transport [5,6]. Also, ELM combines the stripping and extraction processes in a single step [7,8] and is an elaborated form of solvent extraction. Another benefit of using ELM from an environmental viewpoint is the low energy demand compared to pressure-driven membrane processes [9]. In addition, the ELM can be prepared using relatively simple materials and equipment.

The diluent in the ELM process has an important function, since it is the major constituent of the membrane phase and is crucial for its stability and for an effective metal transport. A viscous oil generally increases the stability [10] but has the drawback of decreasing the mass transport. A high density difference between the external phase and the ELM phase would be beneficial for a better phase separation after the extraction, while a low solubility in water is needed to avoid interaction with water that results in emulsion breakage [11]. As a result, the most commonly used diluents in ELM systems are volatile and organic solvents, such as kerosene that has proven to work particularly well for the removal of chromium [5,12,13]. It is of great interest, and a big challenge, to replace such volatile and fossil fuel based diluents with non-toxic vegetable oils. To the best of our knowledge there are very few, if any, reported works exploring the use of a vegetable oil as an alternative to petroleum based solvents in the ELM process. However, vegetable oils including soybean, palm, rapeseed and sunflower oils, have been explored in other types of liquid membranes: *i.e.*, for the removal of phenol [14,15], for Cu(II) extraction [16], for removal and recovery of rhodamine B [17], and textile dye [18]. These studies indicate a great potential for using vegetable oils in chemical processes of environmental relevance. 

In this study we explore the possibility to replace the fossil-fuel based diluent kerosene in the ELM with a non-volatile and renewable vegetable oil, namely food-grade palm oil. The chosen model system consists of an acidic external solution of hexavalent chromium, an alkaline internal solution with NaOH acting as stripping agent, and a membrane phase with palm oil, Span 80, and Tween 80 acting as emulsifiers, and the ionic liquid tri-*n*-octylmethylammonium chloride (TOMAC) acting as carrier. It is important to note that the separation of the organic and the wastewater phase is crucial for a functioning system, however in this study the density difference between the phases is enough to achieve the separation. By studying the extraction of hexavalent chromium, we demonstrate that palm oil can work well as a diluent for ELM systems.

## 2. Experimental

### 2.1. Materials

**Figure 2:**
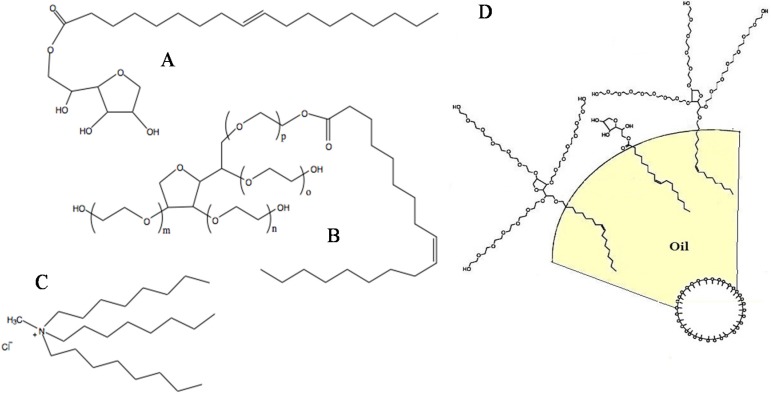
The structural formula of **A**: Span 80 (sorbitan monooleate); **B**: Tween 80 (ethoxylated sorbitan monooleate); and **C**: TOMAC (tri-*n*-octylmethylammonium chloride); **D**: Schematic representation of the geometrical packing of the surfactants at the oil-water interface in dispersed oil droplets.

Palm oil was purchased from the market of brand Buruh, which is cooking oil; a fraction of refined bleached deodorized palm oil called olein consisting of mostly unsaturated fatty acids [19]. Pure Tween 80 was purchased from R&M Chemicals (Petaling Jaya, Malaysia). Span 80, TOMAC, and butanol were obtained from MERCK (Petaling Jaya, Malaysia). K_2_Cr_2_O_7_ (Potassium dichromate) was purchased in powder form from R&M Chemicals. All chemicals were used without further purification. The molecular structure of these components is shown in Figure 2.

### 2.2. Analytical Measurements

The concentration of chromium in the samples was determined using inductively coupled plasma optical emission spectroscopy (ICP-OES) with an Optima 7000 DV ICP-OES from PerkinElmer (Waltham, MA, USA) at the wavelength of 267.7 nm. The device has a dual-view design and a detection limit in the range of parts per billions. The pH of the solutions was measured with a Mettler Toledo Delta 320 pH meter.

### 2.3. Preparation and Evaluation of the ELM System

**Figure 3:**
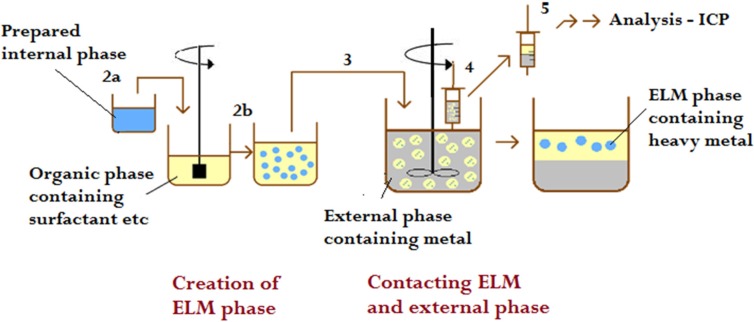
Schematic representation of the procedure of the metal extraction experiments.

The external phase was prepared through dissolving the chromium salt in distilled, deionized or tap-water, and the pH was adjusted with hydrochloric acid (point 2a, Figure 3). The internal phase containing the desired concentration of the stripping agent was prepared with the same type of water as the external phase. The diluent was mixed with emulsifier, co-surfactant, and carrier, while the emulsification of the internal and the membrane phase was conducted using a high speed homogenizer (IKA T25 Digital ULTRA-TURRAX) with an agitation of 3200–3400 rpm (point 2b, Figure 3). The external phase and the ELM phase were contacted using an agitator stirred by twisted impellors, with varying agitation speed (point 3, Figure 3). Samples were taken periodically during each run using syringes left undisturbed for the short time required for the ELM and external phase to separate, and the concentration of chromium in the feed phase was detected (point 4, Figure 3). The concentration of chromium left in the external phase was determined using ICP-OES (point 5, Figure 3).

The removal efficiency (*E*) of chromium was calculated according to Equation (1).

(1) $$E\left(\text{\%}\right)=\frac{C_{0}-\;C_{\text{t}}}{C_{0}}\times 100$$

where *C*_0_ and *C*_t_ (mg L^−1^) are the concentration of chromium initially and at the time of measurement, respectively.

**Figure 4:**
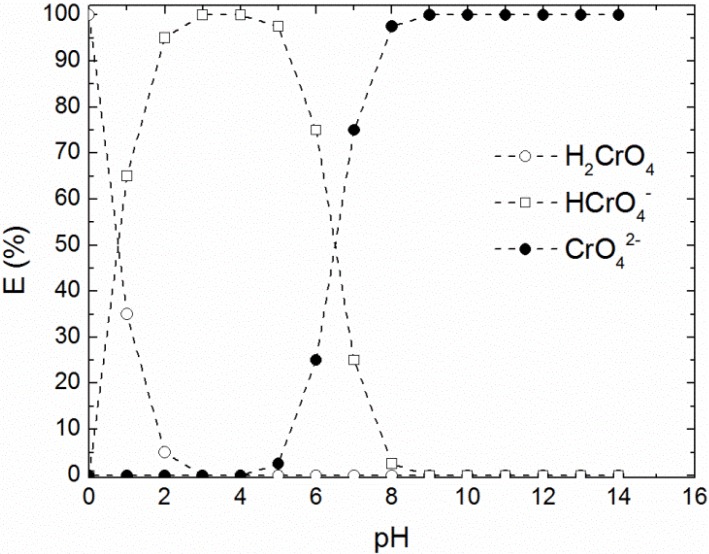
The graph shows the abundance of Cr(VI) ions in water with varying pH. For slightly acidic or basic pH, CrO_4_^2−^ is the dominating form, a further decrease in pH leads to the formation of HCrO_4_^2−^ and H_2_CrO_4_. Data reproduced from [20].

**Chemistry of chromium (VI) and TOMAC**. The species of chromium present in an aqueous solution depend to a large extent on the pH of the solution and the ionic strength. This dependence is graphically shown in Figure 4 for solutions in water. TOMAC was chosen for carrier to achieve an efficient removal since this ionic liquid possesses appropriate properties such as high solubility in the organic phase and low solubility in the aqueous phase, and in addition has previously shown to efficiently extract Cr(VI) [5].

The target complex of chromium is based on the basic properties of TOMAC, thus a low pH in the external phase is required. Due to the presence of the stripping agent NaOH, the carrier exists in two forms: TOMAC (NR_4_^+^Cl^−^) and TOMAOH (NR_4_^+^OH^−^).

**Transport and reaction mechanism**. The reactions involved in the chromium extraction by ELM include the carrier reacting with the stripping agent and the carrier reacting with one of the anionic metal complexes, through an anionic displacement, shown in Equations (2)–(4) below. This transport mechanism is Type 2 facilitation, since the carrier is incorporated in the membrane phase, and in this case it occurs at acidic conditions (pH ~0.5).

(2) $$\text{NaOH}+{\text{NR}}_{4}^{+}{\text{Cl}}^{-}↔{\text{Cl}}^{-}+{\text{Na}}^{+}+{\text{NR}}_{4}^{+}{\text{OH}}^{-}$$

(3) $${\text{HCrO}}_{4}^{-}+{\text{NR}}_{4}^{+}{\text{Cl}}^{-}↔{\text{Cl}}^{-}+{\text{ NR}}_{4}{\text{HCrO}}_{4}$$

(4) $${\text{HCrO}}_{4}^{-}+{\text{NR}}_{4}^{+}{\text{OH}}^{-}↔{\text{OH}}^{-}+{\text{ NR}}_{4}{\text{HCrO}}_{4}$$

The formed carrier-metal complex diffuses across the membrane phase to the internal-membrane interface, where the metal ion is released in the internal-membrane interface and the carrier is regenerated and returned across the membrane as shown in Equation (5). The purpose of using a stripping agent in the internal phase is to trap the metal ion in the internal phase droplets by converting the metal ion into a membrane insoluble compound, *i.e.*, Na^+^HCrO_4_^−^. However, dissociated HCrO_4_^−^ ions in the internal phase will remain in equilibrium after reaction with the hydroxide ions as shown in Equation (6).

(5) $${\text{NR}}_{4}\left({\text{HCrO}}_{4}\right)+\text{NaOH}↔{\text{NaHCrO}}_{4}+{{\text{NR}}_{4}}^{+}{\text{OH}}^{-}$$

(6) $${{\text{HCrO}}_{4}}^{-}+{\text{OH}}^{-}\;↔{\text{CrO}}_{4}^{2-}+{\text{H}}_{2}\text{O}$$

As the stripping reaction proceeds and hydroxide ions are released in the external phase, the pH increases due to exchange of the hydroxide ions with the metal complex. As the pH changes in the external phase, an increased amount of CrO_4_^2−^ ions will be present which consequently react slowly with TOMAC and TOMAOH, see Equation (7). Each CrO_4_^2−^ species requires two extracting species for the reaction with the carrier to occur, resulting in a decreased reaction rate with time.

(7) $${\text{CrO}}_{4}^{2-}+2{\text{NR}}_{4}^{+}{\text{OH}}^{-}↔2{\text{OH}}^{-}+{\left({\text{NR}}_{4}\right)}_{2}{\text{CrO}}_{4}$$

The ion flux through the membrane is created by a difference in chemical potential, which is due to the different pH between the two aqueous phases. It turns out that the factors influencing the performance of the ELM process need to be investigated, wherefore the concentration of the stripping agent, the pH of the external phase, and the concentration of carrier were varied in the initial experiments, in order to evaluate the performance of the ELM system with palm oil as diluent compared to the well-established system based on the use of kerosene.

**Optimization of the ELM system**. A challenge with the ELM process is the instability of the emulsion globules, which is mainly influenced by osmotic swelling and globule breakage. A suitable o/w emulsion formulation of the novel system with palm oil as diluent was required and was formulated through a rough screening, varying the emulsifier concentration and the organic to aqueous phase ratio (*O*/*I*) as defined in Equation (8). The treat ratio, the feed phase (F) to ELM phase ratio, defined in Equation (9), was also explored as it defines the effectiveness and cost of the ELM process. In addition, the effect of water pretreatment was investigated in order to analyze the influence of other ionic species possibly present in the water phases.

(8) $$O/I=\frac{m_{oil\;phase}}{m_{internal\;phase}}$$

(9) $$F/ELM=\frac{V_{external\;phase}}{V_{ELM\;phase}}$$

## 3. Results and Discussion

### 3.1. Chromium Extraction Using the Novel ELM System

**Figure 5:**
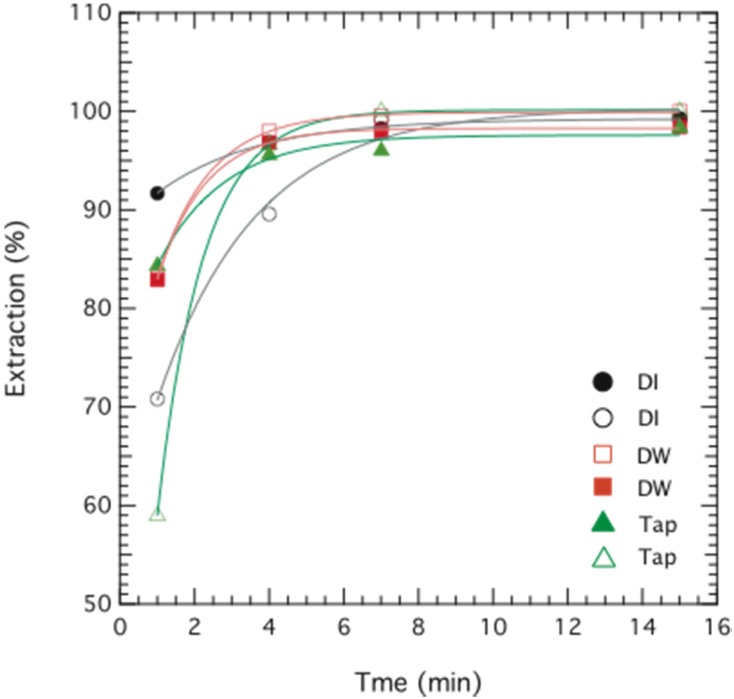
Extraction of chromium as a function of time. Data are plotted for two replicates of emulsion liquid membrane (ELM) formulations containing 2.5 wt% Span 80, 1 wt% Tween 80 and 0.35 wt% TOMAC prepared in de-ionized water (DI; ○,●), distilled water (DW; □,■), and tap-water (Tap ▲,∆), respectively. The initial concentration of chromium was 114 ppm (DI), 95 ppm (DW) and 104 ppm (Tap).

The extraction of Cr(VI) using the novel ELM formulation based on palm oil resulted in 97%–99% removal efficiency after less than 10 min, as seen in Figure 5. This is both high and fast, and is comparable to other studies. Results of Cr(VI) removal efficiency (*E*, %) as a function of time are shown in Figure 5, with the ELM formulations containing 3 wt% Span 80, 1 wt% Tween 80, and 0.35 wt% TOMAC prepared in distilled water (DW), de-ionized water (DI) and tap-water (Tap), respectively. The internal phase contained an optimal stripping agent concentration of 0.1 M NaOH. The observed performance is comparable to that reported in other studies, for instance the study of Goyal *et al.* who observed an optimum removal of 97.5% within the same time range [9], García *et al.* who have reported a removal of 94% Cr(III) within 5 min [6], and Kumbasar *et al.* who could extract 99% Cr(VI) within 6 min [13]. These authors, however, all used kerosene based ELM systems.

Figure 5 also shows that the type of water has a negligible effect on the final removal of Cr(VI), and hence does not influence the ELM formulation, a noteworthy result that points to the robustness of this system. The role of water quality becomes an important factor for the potential use of ELM in a larger commercial and industrial scale, for which it is desirable that the method works for wastewater and is unaffected by the presence of other ions. This was also a reason for choosing a non-ionic surfactant such as Span 80, since the performance of non-ionic surfactants is unaffected by the presence of other ions, as opposed to, e.g., ionic surfactants [21,22].

**Optimization of the novel ELM system**. We find that, as for kerosene, an external phase pH of 0.5 was most beneficial also for the system with palm oil as diluent [5]. However, the treat ratio (*F*/*ELM*) was found to be most favorable when kept at 2 for the palm oil system. When creating the w/o emulsion, the homogenization was kept at 3400 rpm, since a homogenization speed above 3500 rpm (for a solution contained in a 100 mL beaker) resulted in the formation of air bubbles and a highly viscous emulsion unsuitable for extraction. Concerning the stability of the emulsion, a membrane phase with an emulsifier concentration of 2.5–3 wt% and an *O*/*I* = 3 was found to be most efficient.

**Figure 6:**
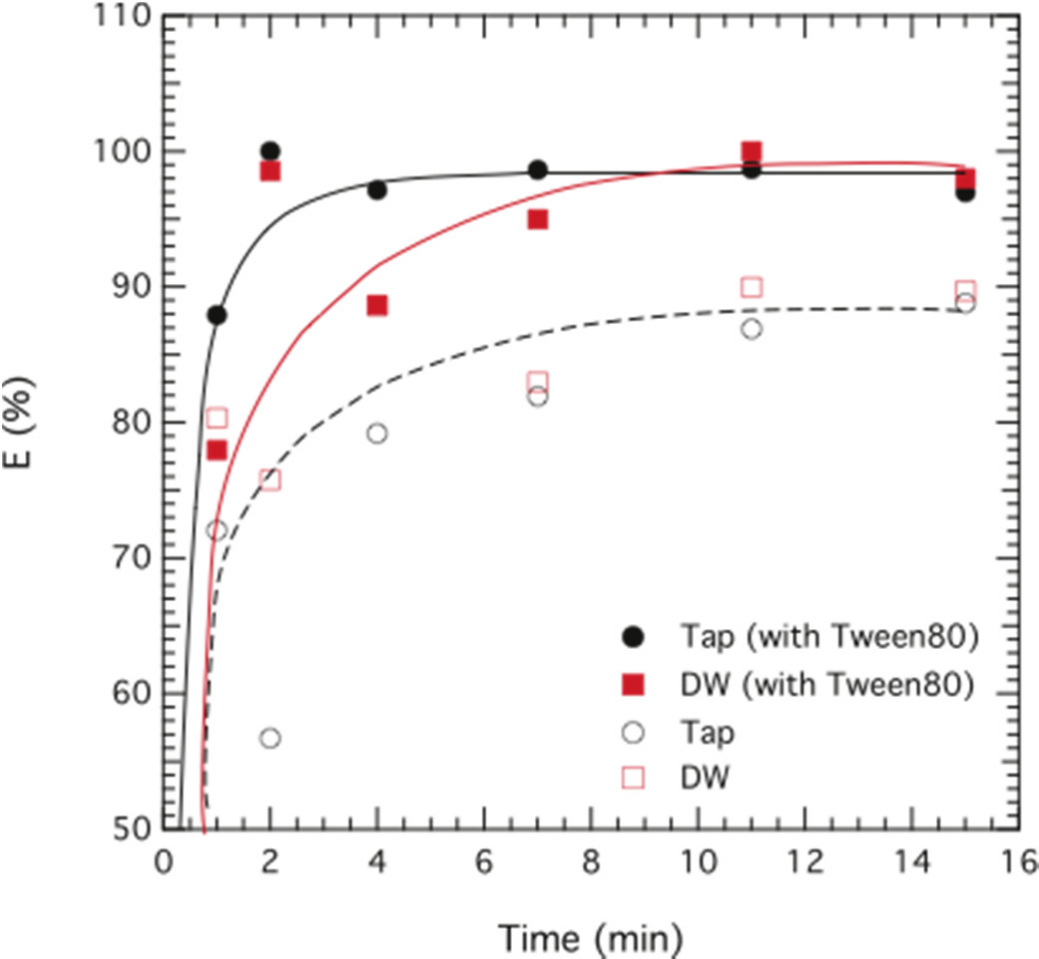
Extraction of chromium as a function of time. Data are plotted for ELM formulations containing 3 wt% Span 80, 1 wt% butanol and 0.35 wt% TOMAC, without Tween 80 (hollow symbols) and with 1 wt% Tween 80 (filled symbols) prepared in distilled water (DW; ■) and tap-water (Tap; ▲). The initial concentration of chromium was 95 ppm (DW) and 104 ppm (Tap).

**The effect of Tween 80**. As a novel approach, the hydrophilic surfactant Tween 80 was included in the emulsion phase. Tween 80 reduces the viscosity of the emulsion and in addition facilitates the creation of a double emulsion during the extraction process. We believe that Tween 80 stabilizes the multiple emulsion, resulting in a more homogeneous solution when contacting the emulsion phase and the external phase, giving a positive effect on the overall chromium removal efficiency, as can be deduced from Figure 6. The use of butanol as a co-surfactant was also applied in some cases since it increased the stability of the emulsion, however it had no significant effect on the extraction rate.

**Contacting the phases**. We observed that when contacting the external with the ELM phase a higher agitation speed (>600 rpm) compared to the kerosene-based ELM (<400 rpm) [9] was needed in order for the solutions to thoroughly mix, and we also visually observed that the use of Tween 80 as a co-surfactant in the membrane phase facilitated the mixing remarkably. Tween 80 is a highly hydrophilic surfactant and should therefore not be soluble in the oil phase of the system. As it is incorporated during the emulsification of the ELM phase, and therefore present at the membrane-internal interface, we speculate that some Tween 80 molecules are transported by microscopically small water droplets to the membrane-external interface, which lowers the interfacial tension and facilitates the second emulsification. In all experiments reported here the treat ratio was kept constant at *F*/*ELM* = 2. The drawback of using Tween 80 is a possible contamination of the feed phase, wherefore a slightly less hydrophilic surfactant may be of interest for future applications.

**Figure 7:**
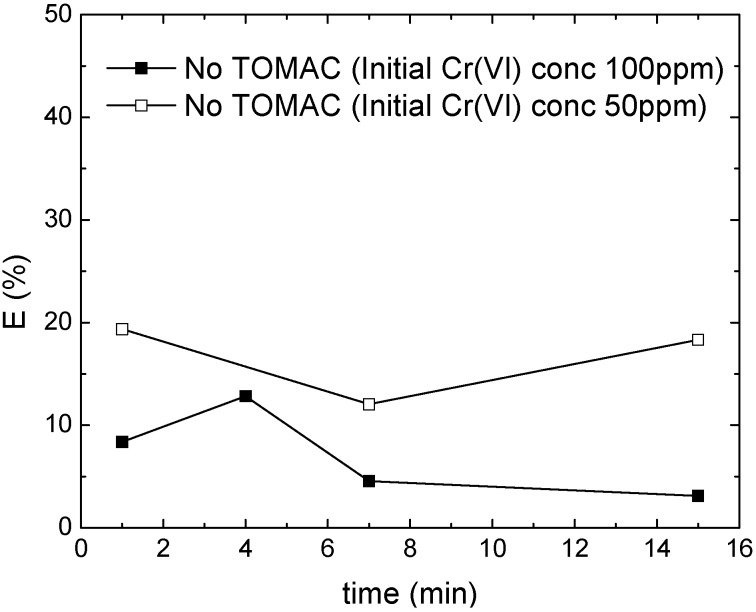
Chromium removal efficiency without TOMAC as carrier. Filled square (■) contains 3 wt% Span 80, 1 wt% Tween 80, 1 wt% butanol and 0.35 wt% TOMAC. Open square (□) contains 2.5 wt% Span 80, 1 wt% Tween 80, 0.35 wt% TOMAC.

**The effect of TOMAC as carrier**. To verify the influence and the function of TOMAC as a carrier in the palm oil based ELM, experiments were carried out with and without TOMAC in the membrane phase. Figure 7 clearly shows that the presence of TOMAC is crucial to achieve a high removal efficiency: the extraction rate observed for systems without TOMAC is decreased by a factor of five to ten compared to systems having the carrier incorporated. These results are comparable to kerosene based systems [5].

**Figure 8:**
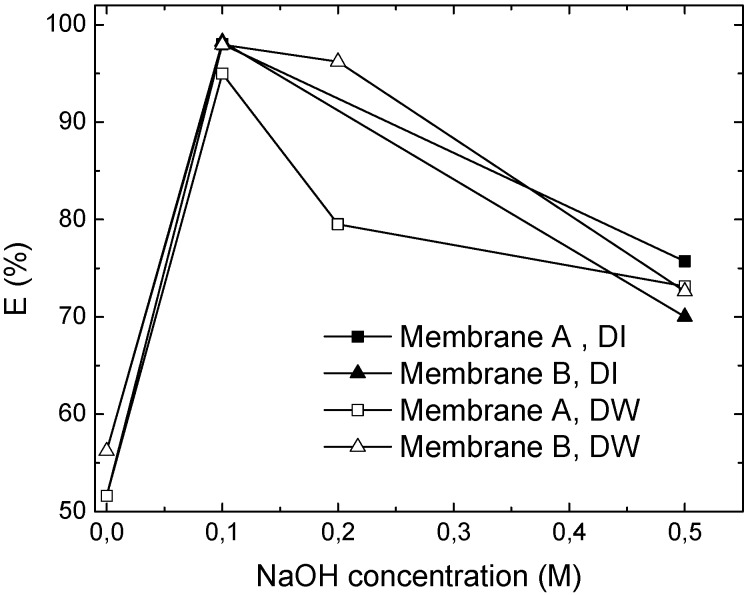
The effect of stripping agent concentration after 7 min. Both membranes contain 2.5 wt% Span 80, 1 wt% Tween 80, 0.35 wt% TOMAC, while membrane A also contains 1 wt% butanol.

**Role of the stripping agent**. A suitable concentration of the stripping agent in the internal phase is one of the important factors in an ELM formulation. In Figure 8 the extraction of Cr(VI) after 7 min is shown as a function of NaOH concentration, demonstrating that the removal significantly depends on the presence of the stripping agent, which in turn should be present at an optimal concentration. This result is consistent with the results of Goyal *et al.* for a kerosene based ELM [5]. The highest Cr(VI) removal is achieved at a NaOH concentration of 0.1 M, while a further increase in the NaOH concentration results in worse performance. At higher concentrations a stronger pH gradient is created, increasing the difference in osmotic pressure and consequently risk of swelling of the internal droplets. The consequent rupture will cause a reduction of NaOH in the internal phase, which reduces the amount of NaOH available for the stripping reaction with the metal complex.

Finally, an optimization study was performed, in which the agitation speed and the concentrations of Span 80 and butanol were varied, while the concentrations of Tween 80 and TOMAC were held constant at 1 wt% and 0.35 wt%, respectively. 

To summarize, the optimal parameters for Cr(VI) removal are 2.58 wt% Span 80, 0.515 wt% butanol, and an agitation speed of 522.6 rpm. The latter is an important factor for the overall chromium extraction process; in fact for higher speeds a more stable emulsion is required. This is achieved by a higher content of surfactant and co-surfactant in the ELM formulation, which on the other hand increases the viscosity of the membrane, in turn requiring a higher agitation speed. The optimization study performed revealed a significant correlation between the agitation speed during the contacting of the phases and the concentration of surfactant, in agreement with the discussion above; at lower surfactant concentrations the viscosity is decreased and lower agitation speeds are sufficient.

## 4. Conclusions

This study demonstrates that the petro-chemically based diluent kerosene can be successfully exchanged for a vegetable oil, namely palm oil, in emulsion liquid membranes. The novel ELM formulation based on palm oil showed a Cr(VI) removal efficiency of 97%–99% within less than 10 min. Even though the treat ratio used in this work may be considered low, together with a somewhat high *O*/*I* ratio for industrial implementations, this work has nicely demonstrated the concept of using a vegetable oil. For real applications further optimizations of the system may be required. When creating the w/o emulsion, a homogenization speed higher than 3500 rpm resulted in an emulsion too viscous to be suitable for extraction. The use of Tween 80 facilitates the mixing and decreases the viscosity of the emulsion thus enhancing mass transport. Despite the high viscosity of palm oil, which might increase the mass transport resistance, we do not observe a less efficient extraction rate. On the contrary, the results show that most of the chromium was extracted within a few minutes. This suggests that mass transport resistance may not be the main rate limiting step and opens up for the possibility of using other vegetable oils with higher viscosities. The stripping agent concentration is important with respect to the emulsion stability, as concentrations higher than 0.1 M NaOH results in decreased removal efficiency. We also observe that the quality of the water (*i.e.*, distilled, de-ionized, or tap water) had no effect on the removal efficiency. This is relevant for the potential of larger-scale or industrial implementations.
